# Items

**Published:** 1878-09

**Authors:** 


### Items.

RESOLUTIONS OF RESPECT.—At a special meeting of the Rockford Medical Association held Wednesday, July 31, the following resolutions were adopted:

WHEREAS, death has removed, after a painful and protracted sickness, our worthy associate and co-worker, A. L. McArthur, M. D. and,

WHEREAS, The members of the Rockford Medical Association desire to express their esteem and regard for his memory, therefore,

*Resolved*, That we have received the intelligence of his death with heartfelt sorrow, and with a deep sense of the loss sustained to the profession.

*Resolved*, That we will cherish his memory with the respect due him as a worthy and active member of the medical fraternity, and also record our admiration of the many good qualities he possessed.

*Resolved*, That we extend our deepest and kindest sympathies to the family, and that these resolutions be forwarded to them as an expression of regard for our deceased professional brother.

*Resolved*, That we will attend the funeral as a society, and that these resolutions be published in the city and Chicago press, and also in the CHICAGO MEDICAL JOURNAL AND EXAMINER.

A. E. GOODWIN, President.

H. W. TEBBETTS, Secretary.

THE following is the programme of the second annual meeting of the American Dermatological Association, held at the Grand Union Hotel, Saratoga Springs, New York, August 27th, 28th and 29th, 1878. Papers: Introductory remarks, by the president, Dr. J. C. White, of Boston; on the pigmentary syphiloderm, by Dr. I. E. Atkinson, of Baltimore; on a new method of permanently removing superfluous hair, by Dr. L. D. Bulkley, of New York; a case of the so-called xeroderma of Hebra, by Dr. L. A. Duhring, of Philadelphia; on the proper use of the term acne, by Dr. G. H. Fox, of New York; a case of scleroderma, by Dr. F. P. Foster, of New York; case of inflammatory fungoid neoplasm, by Dr. L. A. Duhring, of Philadelphia; treatment of hirsuties, by Dr. W. A. Hardaway, of St. Louis, Mo.; epithelium and its performances, by Dr. C. Heitzmann, of New York; idiopathic cutaneous atrophy, by Dr. J. N. Hyde, of Chicago; case of gangrænopsis, by Dr. H. G. Piffard, of New York; the use of linseed and oil as therapeutic agents in diseases of the skin, by Dr. S. Sherwell, of Brooklyn, New York; a further contribution to the study of the xeroderma of Hebra, by Dr. R. W. Taylor, of New York; a case of ulcerative scrofuloderm, by Dr. A. VanHarlingen, of Philadelphia; a case of recurrent cutaneous hæmorrhage with urticarial and bullous efflorrescence, by Dr. J. C. White, of Boston.

The *American Association for the Cure of Inebriates*, will hold its tenth annual meeting at Boston, Mass., commencing Tuesday, September 10th, 1878, at 10 A. M., in "Union Hall." A very important meeting is anticipated.

Poisoned by Kid Gloves!—A merchant recently bought a pair of navy-blue kid gloves in Hamburg. After his arrival in Berlin, he put on the gloves and made several visits. Soon after he felt sick and languid; and a peculiar eruption appeared on the dorsal surfaces of his hands. While searching for the cause of his strange ailing which greatly puzzled his physician, the patient thought of the new gloves and sent them to an experienced chemist for examination. The chemical analysis showed a considerable amount of arsenic in the gloves.—(*Allg. Med. Centralz*).

